# Physical therapists and importance of work participation in patients with musculoskeletal disorders: a focus group study

**DOI:** 10.1186/s12891-017-1546-9

**Published:** 2017-05-16

**Authors:** Nathan Hutting, Wiebke Oswald, J Bart Staal, Josephine A Engels, Elvira Nouwens, Maria WG Nijhuis van-der Sanden, Yvonne F Heerkens

**Affiliations:** 1 Faculty of Health and Social Studies, Research Group Occupation & Health, HAN University of Applied Sciences, P.O. Box 6960, 6503 GL Nijmegen, Netherlands; 2 Faculty of Health and Social Studies, Physical Therapy, HAN University of Applied Sciences, Nijmegen, Netherlands; 3 Faculty of Health and Social Studies, Research Group Musculoskeletal Rehabilitation, HAN University of Applied Sciences, Nijmegen, Netherlands; 4 Radboud Institute for Health Sciences, IQ Healthcare, Radboud University Medical Center, Nijmegen, Netherlands

**Keywords:** Musculoskeletal disorders, Physical therapy, Occupational health, Work participation

## Abstract

**Background:**

Musculoskeletal disorders are a major health problem resulting in negative effects on wellbeing and substantial costs to society. Work participation is associated with positive benefits for both mental and physical health. Potentially, generalist physical therapists (GPTs) can play an important role in reducing absenteeism, presenteeism and associated costs in patients with musculoskeletal disorders. However, work participation is often insufficiently addressed within generalist physical therapy practice (GPTP). Therefore, this study evaluates whether GPTs take work participation into account as a determining factor in patients with musculoskeletal disorders, and how this might be improved.

**Methods:**

This qualitative study consisted of seven focus groups involving 30 participants: 21 GPTs and 9 occupational physical therapists (OPTs). Based on an interview guide, participants were asked how they integrate work participation within their practice, how they collaborate with other professionals, and how GPTs can improve integration of the patient’s work within their practice.

**Results:**

Although participants recognized the importance of work participation, they mentioned that the integration of this item in their GPTP could be improved. Generally, GPTs place insufficient priority on work participation. Moreover, there is a lack of cooperation between the generalist physical therapist and (other) occupational healthcare providers (including OPTs), and the borderlines/differences between generalist physcial therapy and occupational health physcial therapy were sometimes unclear. GPTs showed a lack of knowledge and a need for additional information about several important work-related factors (e.g. work content, physical and psychosocial working conditions, terms of employment).

**Conclusions:**

Although a patient’s work is important, GPTs take insufficient account of work participation as a determining factor in the treatment of patients with musculoskeletal disorders. GPTs often lack specific knowledge about work-related factors, and there is insufficient cooperation between OPTs and other occupational healthcare providers. The integration of work participation within GPTP, and the cooperation between GPTs and other occupational healthcare providers, show room for improvement.

**Electronic supplementary material:**

The online version of this article (doi:10.1186/s12891-017-1546-9) contains supplementary material, which is available to authorized users.

## Background

Musculoskeletal disorders (MSDs) are a worldwide health problem resulting in negative effects on an individual’s wellbeing and substantial costs to society [1]. Common MSDs include osteoarthritis, rheumatoid arthritis, and spine-related neck and back problems [1–3]. Among occupationally active adults, MSDs are the main cause of disability burden [4]. Moreover, people with MSDs face substantial limitations in their activities of daily living, which have an adverse impact on their quality of life [5]. For both the onset and progression of non-traumatic MSDs, the role of physical and psychosocial work exposures is well established [6]. Work-related MSDs are those MSDs which are induced or aggravated by work, including work content, terms of employment, social relationships at work, and working conditions [7]. MSDs represent a considerable financial burden with regard to both direct and indirect costs [8, 9] and these costs continue to rise [10].

Work is associated with positive benefits, including both mental and physical health [11]. Moreover, the longer an individual is out of work due to MSDs, the harder it is for them to get back to work [12]; therefore, early intervention is advocated [13]. In addition, long-term work absence poses a serious risk to physical, mental and social wellbeing, while return to work can improve recovery for individuals with common health problems [11].

In the Netherlands, regular (curative) healthcare is separated from occupational healthcare. Although work participation is an important factor of the perceived quality of life, work is generally insufficiently addressed within regular Dutch healthcare [14]. Also, insufficient knowledge about work-related factors within regular healthcare might lead to a longer duration of both diagnosis and treatment of complaints, and longer absenteeism than necessary [14, 15]. Therefore, the Social and Economic Council of the Netherlands (SER) recommended to enhance the quality of occupational healthcare within the first, second and third-line healthcare, by improving knowledge and by placing more focus on prevention of absenteeism, return to work (work reintegration), and accessibility to occupational healthcare for all employees [14]. Besides occupational healthcare services, also general practitioners and first-line caregivers (including generalist physical therapists [GPTs]) can contribute to better occupational healthcare by paying sufficient attention to work during diagnosis and treatment, and through better cooperation between occupational healthcare and regular healthcare [14].

Physical therapists (PTs) are important for the rehabilitation and return to work of injured workers [15] and can play a major role in the prevention/shortening of absenteeism [16]. There is strong evidence to support the role of GPTs as primary care providers for injured workers with MSDs [15]. The expertise of GPTs in managing work-related MSDs is an important component in facilitating recovery from injury and return to work [15]. As primary care practitioners with appropriate diagnostic skills in MSDs, GPTs are ideally positioned to influence the return to work processes, and facilitate recovery and rehabilitation [15, 17]. Occupational factors influence the extent of sickness absence following an episode of MSDs; however, limited efforts have been made to integrate the identification and management of occupational factors into the routine practice of GPTs [18].

The Netherlands has a specific educational program that leads to becoming a specialist in occupational physical therapy. However, from a total of 19,557 GPTs in the Netherlands, only 169 are registered with the professional Dutch physical therapy association. Therefore, GPTs need to have sufficient knowledge and skills to address the relation between MSDs and the patient’s work, and to refer the patient (if needed) to other professionals (including the occupational health physcial therapist [OPT]). However, GPTs treating MSDs may have some reluctance to include work-related factors in their treatment plan if they lack additional occupational health training [15]. In the Netherlands, it seems that the patient’s work-related factors are insufficiently addressed within GPTP.

Therefore, this study aimed to investigate i) if and how GPTs currently take into account work participation as a determining factor in patients with MSDs, and ii) how the integration of work participation within GPTP might be improved.

## Methods

### Study design and setting

Focus groups were used to investigate how PTs in the Netherlands take into account work participation as a determining factor in their overall treatment (including history taking, physical examination, clinical reasoning and actual treatment) of patients with MSDs, including the experiences, beliefs and needs of the patient. Focus groups can uncover factors that influence opinions, behavior and motivation [19], and provide an interactive environment in which ideas can emerge from the group [19]. A group possesses the capacity to become more than the sum of its parts and to exhibit a synergy that individuals alone do not possess [19]. During March–July 2016, seven focus groups with PTs were held in the Dutch cities of Nijmegen, Utrecht and Breda (HAN University of Applied Sciences or at hotels).

The Medical Ethical committee at the HAN University of Applied Sciences declared that, because the study (Registration no. ACPO 09.01/16) does not fall within the Dutch law on Medical Research involving Human Subjects, no ethical approval was required. The research protocol fulfilled the criteria of the Declaration of Helsinki on Ethical Principles for Medical Research Involving Human Subjects.

### Participant recruitment

During January–July 2016, a convenience sampling technique was used to identify participants who were eligible for participation if they: i) had a physical therapy education, and ii) worked with patients with a paid job and who had chronic (persisting >3 months) MSDs. GPTs, as well as physical therapists specialized in OPT, were eligible for participation. Participants were recruited by calls in newsletters for physical therapists and messages on physical therapy websites in the Netherlands. These calls included some basic background information about the project. There was no relationship established between researchers and participants prior to the study.

All participants gave written informed consent and filled out a short questionnaire (demographics) prior to the start of the focus group. All participants agreed to the audio-recording of the sessions and all received a gift of 60 euro for their participation.

### Focus groups

Following the recommendations of Krueger and Casey [19], we developed a semi-structured interview guide with open-ended questions (Additional file 1). The interview guide was pilot-tested in the first focus group and, because no modifications were necessary, the same guide was used in all seven focus groups. The participants were asked about the way they integrate work participation within their practice, how they work together with other professionals, and how GPTs can be facilitated to (better) integrate work participation within their practice. Each focus group was moderated by the first author (NH); all sessions were audio-recorded and notes were taken by a second researcher (WO, EN). No one else was present besides the participants and researchers. The moderator ensured that every participant was involved in the discussion, and actively generated interaction/discussion between participants. Each of the seven sessions lasted about 120 min (including the introduction to the session). After each session, the moderator and the second researcher discussed the group dynamics and made a summary of the most striking results [19].

### Data analysis

The audio-recordings were transcribed verbatim by the first author (NH). Data were analyzed using theoretical thematic analysis, a method for identifying, analyzing and reporting themes within data [20, 21]. Analysis was performed by taking the following steps: 1) familiarizing with the data, 2) generating initial codes, 3) searching for themes, 4) reviewing themes, 5) defining and naming main categories, and 6) producing the report [20]. The first three transcriptions were analysed by two authors (NH, EN), both trained in qualitative research methods. Thereafter, the codes that initial emerged from the data were compared and discussed until consensus was reached. The subsequent interviews were analysed by one author (NH) and randomly checked by another author (WO). After reading each transcript multiple times, initial codes were generated with an open-coding system [21]. New codes were added when considered necessary. After that, the codes were sorted into themes based on how the different codes are related and linked [21]. Then, the emergent themes were used to organize the data into main categories [21].

The Atlas.ti (version 7.5.13) program was used for coding and managing the analysis, and the supporting quotes related to each theme were discussed by the research group. As no new insights emerged from the seventh focus group, it was considered that data saturation had been reached and that no additional focus groups were required. An email with the draft version of the Results section was sent to all participants with the request to screen the text for misinterpretations and to make additions/revisions if necessary. An email reminder was sent to the participants in case they did not respond to the first email within 10 days.

## Results

A total of 30 PTs participated in this study, distributed over 7 focus groups. The number of participants in each group ranged from 2 to 7 participants (Table 1). Of the 30 participants, 9 had a specialization in occupational physical therapy and 21 were GPTs. Both GPTs as well as OPTs participated in the focus groups (mixed groups). The characteristics of the participating GPTs are presented in Table 1. The mean age of the participants was 37.5 (range 22–65) years and mean experience as a physical therapist was 14.3 (range 1–35) years. Regarding the email that was sent to all 30 participants, 22 responded; however, none of them had any comments on the draft version of the Results section. During data analyses the following main categories were identified: the importance of addressing work; the patient’s own perspective; addressing work participation in the history taking, physical examination, treatment and evaluation; cooperation between GPTs and OPTs; lack of knowledge/skills of GPTs (and how to enhance this), cooperation with other professions, and working together with patients’ companies. An overview of the main categories and themes identified are presented in Additional file 2.

**Table 1:** Demographic profile of the participants

Participant ID number	Gender	Age (years)	No. of years experience as PT	Group	Specializations(s)
1	Female	28	8	1	Shoulder rehabilitation, lifestyle coach, work reintegration and prevention
2	Female	33	10	1	Hand therapy, physical therapy sciences
3	Male	51	28	1	Rehabilitation, hand therapy, neurology, pain
4	Female	31	9	2	Sports PT
5	Male	40	17	2	Manual therapy, ultrasonography, neuro-dynamics
6	Female	33	9	2	Sports PT
7	Male	65	35	2	Sports PT, OPT, manual therapy
8	Female	36	14	2	OPT, sports PT, psychosomatic PT
9	Female	34	8	3	-
10	Male	32	11	3	Manual therapy
11	Male	52	29	3	Human movement sciences, dry needling
12	Male	45	24	4	Geriatric physical therapy, sports rehabilitation
13	Female	27	1	4	-
14	Male	45	25	4	OPT, manual therapy
15	Female	30	6	4	Manual therapy, dry needling
16	Female	29	2	4	Hand therapy, trigger point therapy
17	Female	29	4	4	Manual therapy (student), dry needling
18	Female	31	9	4	Oncology PT, edema PT
19	Female	25	4	5	-
20	Male	39	16	5	OPT
21	Female	50	29	5	McKenzie, Mulligan
22	Male	39	16	5	OPT, master health and social work
23	Female	50	26	5	Neuro-rehabilitation, work reintegration, sports rehabilitation
24	Female	56	33	6	OPT, Hand therapy
25	Female	32	10	6	OPT
26	Female	23	2	7	-
27	Male	47	16	7	OPT, sports PT
28	Male	47	23	7	OPT
29	Female	22	1	7	Physical therapy sciences (student)
30	Female	25	3	7	Human movement sciences

*PT* physical therapist, *OPT* occupational physical therapist

All presented data are based on the opinions/statements of the participants. Each of the main categories is discussed below, with supporting quotations.

### Importance of addressing work

In general, participants found it important to consider and address the patient’s work within GPTP. Because most patients of working age have a job, it is important to take this into account. In some cases, the patient’s treatment goals or expectations are related to return to work or working with fewer complaints and, in those cases, attention to work is often considered normal. Participants agreed that the patient’s work should be part of GPTP, as one participant said:

*I agree that it should be part of it … work is part of the patient’s life. And people have to work till the age of 67 and can be hindered in their work. (participant 4, GPT)*

In case of absenteeism, reintegration should also be within the scope of GPTP. It should be realized that work often represents much more than merely an income; work also provides a type of satisfaction and can make life meaningful. Absenteeism can have a considerable impact on a person’s life. Participants also mentioned that work can be a barrier for recovery, which should be addressed during physical therapy. One participant summarized:

*It’s very important to address this, because work can cause or maintain complaints - that’s one thing. On the other hand, complaints don’t have to be caused by work, but it’s important to know what type of work activities someone is involved in and what the workload is, because complaints can also have an influence on the participant’s work and work capacity. (participant 14, OPT)*

Although all participants agreed that the patient’s work should be a part of therapy, they also mentioned that work is generally not adequately addressed by GPTs and should be more systematically addressed within the clinical reasoning process. Often, GPTs only ask what kind of work the patient does; questioning the patient in detail about his/her work is not done by default. GPTs generally focus more on other daily/sports activities.

Some of the OPTs said that GPTs who are not specialized in occupational health pay insufficient attention to the patient’s work. Participants emphasized that work is a difficult topic to address for many GPTs, particularly when they lack specific knowledge. One participant said:

*I think that if you have insufficient knowledge of occupational factors, or you don’t have affinity with the subject, you won’t address these factors sufficiently. (participant 6, GPT)*

A wider perspective, including the patient’s work and employability, was considered important. Detailed knowledge of which activities are necessary to perform the work might help explain the cause of the complaints and provide more insight into the complaints.

### The patient’s own perspective

According to the participants, patients have a need to address their work within GPTP. Some patients mention work spontaneously and have a need for information, e.g. with regard to the moment they are allowed to work again in case of absenteeism. According to the participants, most patients seem to understand why the occupational health physical therapist GPT also asks questions about their work and work situation. However, because not all patients see a relation between their work and complaints, GPTs have to explain to them why they need to ask questions about their work. One participant said:

*… and you have to ask yourself to what extent the patient is aware of the influence of his/her work on their complaints, or the complaints on his/her work. (participant 4, GPT)*

Participants mentioned that, when patients are unaware of the relation between their disorder and their work, it can be difficult to discuss work and work-related factors; this might require a lot of explanation, whereas the relation with, e.g. sports, is often clearer for patients. If patients feel they have limitations in their work activities, then work is often also a part of their treatment demand and expectations. Also, some patients do not expect a work-related intake; they just want to be cured quickly. GPTs also notice some resistance with regard to discussing work, e.g. in individuals with a labor conflict or who derive some benefit from being sick, or employees who are afraid of being fired because of their absenteeism. However, this resistance can also occur in relation to other factors in the private sphere. Moreover, when the complaint is not directly related to physical factors at work, but more related to psychosocial factors or a barrier for recovery, participants experience that patients are less open for these factors compared to simple physical factors. Also, immigrant patients may have different health beliefs which can hinder discussions about work-related factors; it may take some time to create a safe environment to discuss these factors.

Although many participants ask questions about the patient’s work, this is highly dependent on the presentation and expectations of the individual patient. Mostly, the demand for care is the main item for physical therapists (PTs). Generally, the demand for care is simply related to having less/no pain or wanting to participate in sport activities/household tasks, so that work is not necessarily included in a systematic approach. One participant said:

*I think it really depends on the patient’s demand for care. I always try to translate the demands of care into the main treatment goal. And if you see work-related factors as a barrier for recovery and the patient doesn’t, then you should make it very clear why you want to add that as a treatment goal. (participant 5, GPT)*

### History taking

Participants mention that the patient’s work should be a part of history taking. It is considered important to investigate whether or not work is involved as a causal factor or a barrier for recovery. The attention paid to work varies between GPTs and is also related to the type of patient. Although all participants include work in their history taking, the extent varies between therapists. One participant said:

*Many colleagues question their patients about what kind of work they do, but don’t ask exactly what the work consists of - how many hours? - do you like your work?* etc. *… (participant 22, OPT)*

Some participants ask the patient about work content (e.g. alternating tasks, posture), physical and psychosocial working conditions (e.g. working atmosphere), terms of employment (e.g. working hours), and social relationships at work. These items were generally endorsed as being important by the other participants. With regard to working conditions, one participant said that, for example, a good chair is important, but you have to adjust it and use it properly. Another participant replied:

*… if you have tight deadlines, even if you have a really good chair - that won’t reduce work stress… (participant 21, GPT)*

Load and capacity were also mentioned as important topics. Discussing possible alternative working activities and other solutions may result in less absenteeism. Also, discussing work stress, assistance at work and work atmosphere, was considered important. Participants emphasize to focus not only on physical factors, but also to consider behavioral and psychosocial factors. Asking about work satisfaction was considered important because this can influence or even cause the patient’s complaints. Sometimes participants feel that discussing the more psychosocial factors and job satisfaction can best be done after a few sessions, when people feel more at ease and safe. It was also mentioned that somatization is a ‘trap’ in physical therapy treatment, especially in the population with (long-term) absenteeism. Work-related factors are very diverse and focusing only on the physical complaints/characteristics of the workplace (adaptations and tools) can sometimes result in somatization.

After history taking it is important that GPTs have good insight into the patient’s work and work activities. Focusing on problematic work activities is often helpful. It is important to gain insight into all the work activities and physical load of the patient, which can vary substantially between patients. One participant said:

*When someone is working behind a desk the whole day, it’s possible to imagine his working activities. (…) But sometimes you have no idea about someone’s activities - So you really have to ask someone to describe and show their activities (…) to really understand what someone is physically doing eight hours a day. (participant 1, GPT)*

Participants agreed that work should be addressed specifically and not in general. Although most participating GPTs ask patients about their work, many feel that they could do this better and that, especially their colleagues, could pay more attention to the patient’s work:

*I ask the patient a lot… how many hours do you work and what kind of work? Do you have desk work, do you also walk (…) - a lot of things. But perhaps that’s the reason why we’re here, because we already pay a lot of attention to those factors… (participant 23, GPT)*

Another participant reacted:

*Yes, you’re really a shining example, because my colleagues definitely don’t do that. (participant 22, OPT)*

#### Electronic patient’s file

The electronic patient file (EPF) was mentioned as a major facilitating factor when addressing work within GPTP. If work is included in the EPF, GPTs are stimulated to address work. Participants state that work does not play a major role in the EPF, sometimes it is only mentioned as a barrier for recovery. One participant stated:

*It would be a great idea for the developers of the EPF to include these work-related factors in a better way. (participant 19, GPT)*

Also, including work-related guidelines in the EPF was considered important. However, participants mentioned that completing an EPF takes up valuable time. Moreover, work is not the only topic that should be addressed; however, there is insufficient time to extensively discuss all these topics.

#### Questionnaires

Some OPTs use questionnaires, e.g. the Back Check, which was not used in GPTP. Therapists do not want to burden their patients with too many questionnaires. GPTs sometimes use questionnaires to make an estimation of yellow flags and barriers for recovery. Most GPTs use the Patient-Specific Functional Scale [22] (because it is short/easy to complete) on which patients can also fill in work-related activities. Some participants are aware that the Disabilities of the Arm, Neck and Shoulder (DASH) questionnaire [23] also has an additional work module, but were not using it. One participant used the Traffic Light model for Physical Load in case of Visual Display Unit work; another used the Work Reintegration Questionnaire [24]. Many participants were not aware of the existing work-specific questionnaires. Some participants use online questionnaires, e.g. to measure work stress. Participants also mentioned that standardized questionnaires are sometimes difficult for patients to fill in. One OPT, who used questionnaires, stated:

*For example, questionnaires about the work place, or physical factors, how they deal with complaints, taking breaks, those kinds of things. They have to fill them in and then also think about those things in advance… (participant 24, OPT)*

Participants do see possibilities for questionnaires and checklists. Integrating questionnaires and screenings lists in their practice was recommended (e.g. with regard to expected chronic absenteeism, related factors, posture, workstyle). Questionnaires, which can be filled in online, can also be sent before history taking; they can be used to investigate possible relationships between complaints and work, listing the psychosocial factors such as work pressure, and assessing the risk of chronic complaints or chronic absenteeism. Moreover, questionnaires could be used to indicate whether a specific patient should be referred to an occupational physical therapist (OPT) or other healthcare professional. There was a need for an overview of existing work-specific questionnaires and it was recommended to develop questionnaires with regard to work-related factors, if they do not exist.

### Physical examination

In addition to questions in the history taking, some participants ask their patients to describe their working activities to gain insight into their daily work. Patients can be asked to bring work equipment (if possible) to the practice, or are asked to demonstrate their activities, or the GPT can observe their posture. One participant used myo-feedback; some OPTs use a functional capacitation evaluation.

One participant said that during the first session she did not focus on work but more on body functions, also because of limited time. In the second and third sessions she tried to examine simulated work situations; this approach was recognized by more participants. One participant said:

*I always do the physical therapy examination first. And then I investigate how people perform their work, end-range of motion, unfavorable or static postures, repetitive movements - and then I consider whether their complaints might be caused by their work activities. But first I need to know what their complaint is. (participant 24, OPT)*

#### Workplace investigations

OPTs have experience with workplace investigations, whereas GPTs have limited experience with this. One participant gave the example that someone from the technical service had conducted the workplace investigation, instead of a physical therapist (PT). Another participant reported that a patient mentioned that he has an ‘adapted’ chair, but it proved to be a chair that was adapted for another colleague. Another participant said:

*Recently I visited a hairdresser who had neck and shoulder pain - so I observed her. She’s cutting, cutting, cutting and suddenly she stops cutting and asks the customer how her children are doing -but she held her arm and scissors up in the air. It’s important to notice that… (participant 3, GPT)*

Participants agreed that having the possibilities and skills to perform a workplace investigation is a great advantage. Even though employees may have a totally adapted workplace, how they use it and how they behave there can be something different. Observation of working behavior can also be part of a workplace visit/investigation. For GPTs it is difficult to perform a workplace investigation because they lack the time/skills for this, and it is not covered by the patient’s health insurance. Some OPTs mentioned that a workplace investigation might not be the domain of the GPT, because it requires a high level of knowledge/skills and the results must be useful for the occupational health physician or in future procedures.

Visiting the workplace provides much more insight. Generally, GPTs have a very practical (often a ‘hands on’) approach. However, with regard to the patient’s work, an ‘eyes on’ approach is also important. Observing the patient’s work situation, if possible, provides insights that could lead to adjustment of the treatment. One OPT said:

*If I visit a company, I sit next to the patient for 15 min. I tell the patient that I’m writing down some things - and then I observe (…) Then at a given moment you’ll see that they fall back into their old behavior. That’s something you won’t see in the first 5 min. (participant 25, OPT)*

Some participants made use of pictures and (video) movies; this can be done by the patients themselves, or by colleagues, even at an unexpected moment. This is considered a good way to get an initial impression of the patient’s workplace.

### Treatment

Participants mentioned that asking about work-related factors is of no use unless these factors are addressed in the subsequent goals and treatment. When the patient’s needs/expectations formulated in the demand for care are related to work, participants include work in the therapy goals (mostly in the main goal). Sometimes work is also included as a barrier for recovery. If work is not part of the demand for care, participants sometimes include work as a sub-goal. Then, fulfilling the sub-goals will lead to fulfillment of the main treatment goal. One participant said:

*I include work within the main goal:* e.g. *wants to work 40 h again. Then I continue with the sub-goals - with muscle strength, endurance, flexibility. So that’s an application point for the PT. These are the practical things that he needs for his work, those are my sub-goals. (participant 3, GPT)*

Some participants said they had the impression that the goal with regard to work sometimes vanishes, because one focuses on other goals and, therefore, work gets sidetracked. One participant (who had experience as an auditor) said that in many cases he had not seen goals related to work, or functional treatment related to work activities, even when it was obvious that the factor work was involved.

#### Workplace adaptations

When a workplace investigation had taken place, workplace adaptations were sometimes carried out by OPTs or advised by GPTs. This was not always the case, as one participant said:

*I often hear that advice was given and workplace adaptations were carried out by the technical service of a company. (…) I think that’s strange. (participant 25, OPT)*

Participants emphasize that those adaptations can also result in or stimulate somatization. Moreover, it was stated that very limited evidence exists to support these ergonomic adaptations.

#### Advice

Providing advice and information is considered an important part of PT practice, especially in occupational physical therapy practice. Advice/providing information was even considered as important as other forms of therapy, such as massage, mobilization, or exercises. It was also considered important to provide insight into the relationship between work and complaints, and to create awareness with regard to possible risk factors and solutions.

Giving advice to patients about work-related factors was considered important. However, the level of confidence in providing good advice varied between participants. Participants give advice with regard to posture, workspace, load and capacity, and taking breaks. Providing information and advice may include self-management strategies, as one participant said:

*I always say to patients: at the last treatment, I hope that I provided you with a sort of toolbox with all kinds of things you can use in case of recurrent complaints. Just pay a little more attention to that, take some more breaks. I didn’t have time to deal with sports activities, I can treat that trigger point myself… That’s the way I try to work. (participant 24, OPT)*

In fact, in many cases it is all about facilitating a behavioral change. However, that takes time and people have to work on their behavior themselves. It is important that the patient has enough insight and knowledge with regard to his/her complaints and work load, and has enough practical skills to actually change the behavior. If someone works 40 h/week in hindering conditions, 30 min treatment per week will not be sufficient to help. Facilitating self-management also involves communication skills for discussing things with their supervisor, employer and/or other professionals. One participant said that one could say that the GPT gives the patients part of the responsibility in their treatment. For GPTs it is important to have sufficient knowledge of the patient’s work situation, as one participant stated:

*I think that if you’re not experienced within the domain of work it’s difficult to give good advice. Of course you can advise two weeks of rest, that’s not difficult - but to provide advice specifically about someone’s work situation is difficult - then you really must have sufficient insight in the work processes. (participant 8, OPT)*

#### Functional training

In their treatment, some participants include functional training specifically targeted at work activities, and some noticed that they increasingly integrate functional exercises. Although they try to simulate the work situation as much as possible, it is better to practice at the work site itself. One participant said:

*It’s best to train people at their workplace or at their home. To make it as functional as possible.(…) We have a screw plate, but people say:”… If I’m working on a car then somehow I always have to get around a corner”. So, it’s always different if you try to simulate the situation - it’s never the same as their actual work. (participant 2, GPT)*

For GPTs it is difficult to visit their patient’s place of work. Therefore, some participants said that they try to stimulate their patients to use specific exercise in their work environment. Often theoretical things (like lifting techniques) do not work in specific work situations. Therefore, it is also important to have insight into the exact working situation. One participant said:

*… a man working in agriculture with that tree, that’s something really different (…) that demands some creativity from the physical therapist. What activities do you perform at work? Can you show me? - and then you have to be creative about offering functional exercises, or maybe you need to forbid him to lift things… (participant 8, OPT)*

Other examples of farmers, window washers, plasterers, nurses, childcare employees, and others were given. One OPT said that GPTs need specific knowledge to simulate the work situation. Another participant said that training of work-related physical capacity is difficult and is not often done by GPTs. One participant gave the following example:

*If you want someone to play volleyball again, you can imagine what skills are needed. Even if I don’t play volleyball myself, I can imagine what I need to do. That’s the same with work… (participant 21, GPT)*

### Evaluation

When work is part of the main goal or one of the sub-goals, participants evaluate work in the follow-up, or when they finish their treatment. However, when questionnaires were used and the patient does not report any problems, these questionnaires are not often used again in the evaluation. With regard to the use of questionnaires one participant said:

*I see some major disadvantages in the use of evaluative questionnaires in general, because patients often find it difficult to fill in these questionnaires. That’s also the case with questionnaires related to the patient’s work. (participant 1, GPT)*

In the evaluation of the therapy, the number of working hours should also be taken into account. If a person works more hours, but with the same score for pain, then there is still some progress. Sometimes it is necessary to explain this to a patient.

### Cooperation between GPTs and OPTs

Cooperation between GPTs and OPTs was considered important. After evaluating the factor work as a cause of the complaints and as a barrier for recovery, the problem can be addressed by the GPT, or the patient can be referred to an OPT. One participant explained that he had little confidence in working together within a GPTP in general, he said:

*Too often I see that colleagues think that they’re very good, and that - for example - a sports injury is treated by a GPT even if a sports physical therapist is in the room next to him. And that’s the same with manual therapy and OPT. (…) If that basic attitude doesn’t change, good cooperation between a highly specialized therapist and the GPT will never become a reality. (participant 8, OPT)*

Some participants recognized this viewpoint, whilst others mentioned that this varies between different private practices. One practice has the rule that, in case of a recurrent problem, the patient will be seen by a colleague OPT. Also, if a GPT sees a work-related component the patient will be referred; however, this does not often happen.

Most participants mentioned that cooperation between OPTs and GPTs could be valuable. However, most GPTs find it difficult to establish collaboration with an OPT. It is easier to cooperate if an OPT is available in the practice location. It is also easy to discuss a patient in a more informal way and, if an OPT is available, the work is more within the scope of the GPTs within that practice. However, many participating GPTs do not work together with an OPT in their vicinity and never refer a patient to an OPT. Sometimes they refer the patient back to the general practitioner, mostly in case of insufficient recovery and if the GPT has no treatment options left. Therapists who have an OPT within their own practice do work together with the OPT and experience this as added value. However, one participant who had an OPT in her practice never referred her patients to this colleague. One OPT said:

*In our practice, my qualities are almost never used… (participant 22, OPT)*

This was recognized by some other OPTs. Participants mentioned that, if a patient is referred to an OPT, this often takes place when therapy is not successful, but not in an early stage of the treatment. GPTs generally agreed that they are often too late in referring their patients. Patients should not only be referred after recurring problems or disappointing treatment results, but also (if indicated) at the beginning of an episode. Sometimes GPTs find it difficult to refer a patient to another practice; however, mostly they do not know an OPT in the vicinity. One participant said that they have a referral list for all kinds of problems and specializations, but that the list lacks an OPT. Knowledge about the OPT and referral could be enhanced by forming regional networks with OPTs and GPTs Moreover, not many OPTs are available and not every OPT is registered with their professional association.

#### Differences between generalist physcial therapy and occupational physical therapy

For most participants the borderline between generalist physcial therapy and occupational physical therapy was unclear. Participants mentioned, for instance, that GPTs lack knowledge about OPTs. For GPTs it is often unknown what the scope of the OPT is. One participant stated:

*I have no idea what an OPT does that differs from a GPT in private practice… (participant 9, GPT)*

This idea is widely shared among GPTs. However, this is not only the case with regard to the OPT, but with regard to other PT specializations. A difference between the GPT and OPT is that GPTs generally treat patients in their private practice; patients are questioned there about their work. The OPT is able to visit the workplace and perform a workplace investigation. One participant explained:

*As GPT you work with people, but you don’t visit their work. You question them about it and you explain - How about your workplace? - that kind of thing. But if you really want to work with them, you have to visit their workplace and that’s something that‘s not done by GPTs. (participant 26, GPT)*

Some participants mentioned that it would be valuable if they could make better use of the OPT’s knowledge. It was mentioned that OPT and GPT can also work together such that the GPT treats the functional problem and the OPT can focus on the work-related factors and provide specific advice and interventions at the patient’s workplace. OPTs could have a type of consulting function for GPTs.

If work is the cause of the complaints, or worsens the complaints, then an OPT should be involved. However, GPTs often overlook work as causal factor and do not have the skills to examine work as a factor in the onset/persistence of complaints. Also, in case of blue or black flags, referral to an OPT can be appropriate. However, first of all a GPT has to ask the right questions to confirm these flags, which is not generally done. If work is only considered as a co-factor, a GPT can often handle the complaints himself (although that was also considered to depend on the knowledge/skills of the individual GPT). There is some difference between participants with regard to work-specific exercises. Some GPTs are of the opinion that GPTs are able to provide these exercises, whereas some OPTs argue that to simulate specific work situations specific knowledge is necessary. However, one GPT said:

*For treating work-related complaints - maybe this sounds arrogant - but I don’t think I need any additional skills. (participant 9, GPT)*

For both GPTs and OPTs it was considered important that they cooperate with other professionals and that each professional focus on his/her own area of expertise. It was also necessary to have sufficient knowledge about the expertise of other (occupational health) professionals.

OPTs have specific knowledge, especially with regard to how things work within companies and about laws and regulations. Also, the occupational health language is different from the PT language. One OPT explained that, as a PT, although you only see one patient, that person is part of a company with interactions, culture, colleagues, etc. A value of the OPT might be that he/she has more knowledge and experience with these kinds of factors. Furthermore, it is highly dependent on the interest of the GPT whether or not he/she has sufficient specific knowledge.

Most GPTs (and OPTs) agreed that, because GPTs know little about the specific knowledge of the OPT, they might think that they can address work-related issues themselves. The OPT can act on a broader spectrum; they are also skilled with regard to the organization of work, work tasks, work hours/duration, and work pressure. The following quote from a GPT reflects the generally shared desired situation:

*On the one hand, you want the GPT to be able to notice and address work-related factors, certainly more than is done at the moment. I think we have the possibilities to address these work-related factors to a greater extent within GPT practice. On the other hand, you need to clearly realize your own boundaries – and at what moment do I need to refer to the specialist? (participant 5, GPT)*

### Lack of knowledge/skills of GPTs

In the opinion of the participants, GPTs generally have insufficient specific knowledge to address the patient’s work. GPTs should pay attention to work-related factors and have basic knowledge/skills with regard to addressing these factors. GPTs should assess whether they should refer a patient to an OPT, preferably in the first session. Therefore, a focus on work and sufficient knowledge is considered necessary. One participant said:

*I think it’s impossible to transform every GPT into an OPT - I think we should leave that specific expertise with the OPT. One should focus on basic knowledge for general advice and better referral to specialists. (participant 29, GPT)*

Knowledge about the possible relationship between work and complaints, and how that can be evaluated, was considered important. Participants mentioned that GPTs need knowledge on the following areas: work content (e.g. work analysis, work tasks, posture, lifting, work load, time management), physical and psychosocial working conditions (e.g. working atmosphere, stress management), terms of employment (e.g. working hours, absenteeism), and social relationships at work (e.g. personal factors, communication skills). These factors may be barriers for recovery. Moreover, knowledge about other healthcare providers, laws and regulations, and the occupational language was considered important. With regard to laws and regulations one participant said:

*I think laws and regulations are very important. (…) That you know how to deal with that - not only with regard to obligations, but also concerning the rights of the employee/patient. That could be an important factor. (participant 5, GPT)*

Another participant added:

*I also find it difficult to know my own responsibilities and boundaries as PT. (participant 4, GPT)*

It was considered very important that GPTs not only have the knowledge/skills to address work in the history taking, but that they are also able to intervene with regard to work-related factors and have a network to refer to.

#### Courses

Participants mentioned that they never choose courses with regard to work; also, only a limited number of courses are available. The participants choose courses on, e.g. relaxation exercises or shoulder problems, which are mainly physical therapy-related subjects. However, although many occupation-related subjects are important for GPTs, they receive no education for them. Work could also be included as a topic in seminars; these courses should also include practical aspects, e.g. visiting a work site. Some of the participants said that they would be interested in these courses, one participant said:

*I would be interested in such a course. Not immediately a master’s or another type of education, but a course to upgrade my knowledge and skills. (participant 4, GPT)*

#### Educational programs

Participants mentioned that in their GPT education, work was either not addressed or not addressed extensively; this applied to both older and younger GPTs. However, nowadays, focus on work in educational GPT programs varies between the different universities of applied sciences. In some programs it is part of the basic education, but is often not treated explicitly. Sometimes it is possible to follow a minor module about work. Participants also considered it important to address work in all educational programs leading to GPT specialization. One participant said:

*In my GPT education ‘work’ played no role at all. For me it’s clear that there’s a lot of ignorance about work, which might result in non-optimal treatment. (participant 17, GPT)*

#### Other facilitators

Another suggested way of improving GPTs’ knowledge is a buddy system or peer support in which GPTs discuss their patients with a colleague based on the patient’s EPF. Also, including work and work-related factors within guidelines can improve the way that GPTs address work.

Participants also mentioned that there are many relevant sources of information, but these are often not known by GPTs. Information about work, as well as the guidelines of other healthcare providers, can be very useful. It would be valuable to have an overview of all these sources. In one focus group an app for GPTs was discussed as a source of information. One participant said:

*You could think of an app in which you integrate everything - tools, questionnaires, guidelines, information about laws and regulations. Then work really is the central factor and you can use it as a valuable source. (participant 5, GPT)*

Such an app was considered useful, as were videos and knowledge clips, as they could help GPTs to focus more on work and work-related factors.

### Cooperation with other professions

#### Occupational health physician

Most GPTs have limited contact with occupational health physicians. On the other hand, cooperation between the occupational health physician and OPTs was much better, especially with OPTs working within a company. Some contact takes place via telephone or mail between PTs and the occupational health physician. One participant had more contact with an occupational health physician because that physician happened to rent office space in her private practice.

Another participant added that the initiative always lies with the GPT and that, sometimes, he feels as though he is not taken seriously; this opinion was shared by other participants. One participant said that this might be because the GPTs have limited knowledge about occupational issues. Another participant said:

*There is no or little communication with occupational health physicians. I have the idea that you’re not allowed to interfere, you’re not allowed to say something. And in my opinion, occupational health physicians give the patient the impression that they will inform the GPT - but, in the nine years that I’ve been working, this has seldom happened. (participant 6, GPT)*

The accessibility to, and communication and cooperation with occupational health physicians was considered far from optimal. However, participants said that cooperation between occupational health physicians and GPTs is essential, e.g. to assess the patient’s functional capacity. The information collected by both professions is considered important for the other profession. One participant said:

*I think a lot of benefit is possible through better cooperation with occupational health physicians. Really helping the patient - instead of everyone focusing only on their own part. (participant 10, GPT)*

Patients often ask the GPT for advice about their appointment with the occupational health physician; however, most GPTs are very careful about giving such advice to their patients. Alignment between occupational health physicians and PTs, especially with regard to the return to work policy, was considered very important.

For some participants the role/function of the occupational health physician was not clear. Nevertheless, knowledge about this role was considered crucial with regard to professional cooperation. However, GPTs also think that the knowledge of occupational health physicians with regard to GPT and occupational health physical therapy is also insufficient/lacking. Moreover, GPTs do not always speak the ‘occupational health’ language of the occupational health physician.

#### Occupational therapist

Some participants work together with an occupational therapist. One participant had an occupational therapist working in their practice. One OPT who worked in a multidisciplinary center said that, in that setting, the occupational therapist performed the workplace investigations. Another OPT said that, when patients have limited insurance coverage for physical therapy, she refers them to an occupational therapy practice for a workplace investigation. Although parts of the work of the occupational therapist and OPT overlap, e.g. with regard to workplace investigations, there are also major differences. For instance, with regard to workplace adaptations and devices, (O)PTs focus more on recovery, taking breaks, training, etc. One participant said:

*I think our strength lies in making a link between complaints - and the nature of the work activities and the training of work activities. That’s something an occupational therapist doesn’t do. They don’t perform a physical examination in the same way that we do. (participant 24, OPT)*

#### Other professionals

Some participants work together with a psychologist, mostly after referral by their general practitioner. Especially when yellow flags and psychological/psychosocial factors are present, patients are referred. It is considered important to be aware of the limits of one’s own expertise, and to refer if necessary.

OPTs mentioned that they often work also more closely with human resource staff, occupational health specialists, and case managers.

### Working together with companies

Participants think that GPTs should have more cooperation with companies. GPTs and OPTs can play a greater role within companies, especially with regard to prevention. Such cooperation can exist by having a GPT at the company location, or by referring employees to the GPT practice. Although it was considered difficult to get ‘into’ a company as an individual GPT or even as an OPT, some participants succeeded in setting up a cooperation with one or more companies. OPTs working in private practice sometimes work together with certain companies, or make price quotations for workplace investigation of an individual employee. GPTs think that they can play a role by offering interventions with regard to work and also with regard to prevention; however, this might be the scope of OPTs. Some participants mentioned that there is evidence for the beneficial effect of some interventions and that GPTs should be more proactive.

## Discussion

This study evaluated whether and how GPTs in the Netherlands take into account work participation and work-related environmental factors as determining factors in patients with musculoskeletal disorders and how these could be improved. In general, participants found it important to address the patient’s work within the GPTP because this work can be a causal factor, or a barrier for recovery, or the complaints can influence the patient’s work. However, although participants recognized the importance of this, they mentioned that work participation should be better integrated within their practice. GPTs working with patients with MSDs seems to focus more on bodily functions and limitations, and less on activities and participation, especially participation in work. Participants mentioned that GPTs put insufficient focus and priority on work-related factors, and that work is not always included in the scope of GPTs.

This study revealed that a major issue to be addressed is the lack of cooperation between the GPT and (other) occupational healthcare providers, especially with the OPT and the occupational health physician. Although cooperation between the GPT and the OPT was considered important, very few participants cooperated with an OPT or, in the case of the OPT, cooperated with a GPT. Moreover, the borderline between generalist physcial therapy and occupational physcial therapy was unclear and, in some cases, the differences between generalist physcial therapy and occupational physcial therapy were also unclear. It was emphasized that OPTs have specific knowledge and that occupational physcial therapy is a valuable specialization. However, some GPTs mentioned that they were able to address most of the work-related factors themselves. This study supports the idea that facilitating cooperation between the GPT and the OPT is necessary [14].

Given the advice of the Social and Economic Council of the Netherlands to enhance the quality of occupational healthcare within first, second and third-line healthcare by improving knowledge, and placing more focus on prevention, return to work (work reintegration) and accessibility for all employees [14], it is important that all GPTs address the patient’s work within their practice. Moreover, a considerable proportion of health-related productivity loss is attributable to presenteeism, i.e. decreased work performance while at work [25–27]; this is reported to play a greater role in lost productivity than sickness absence [27]. In the present study, participants agreed that GPTs should have sufficient knowledge/skills to properly investigate the association between the patient’s complaints and work, and the influence of the complaints on the patient’s work. It would be valuable if GPTs could (to some extent) address these factors in their practice; however, this can vary between GPTs depending on their experience, education and interests. Because few GPTs have specialized in OPT, GPTs should at least have sufficient knowledge to be able to refer patients to an OPT or another occupational healthcare provider.

Participants emphasised that superficially addressing work during history taking is insufficient; patients need to be questioned about their work, including all work features and psychosocial factors. These psychosocial factors are known to be a major barrier for return to work [17]. Moreover, it was considered important to address work-related factors in the subsequent treatment goals and treatment.

In the present study, the patient’s attitude was mentioned as an influencing factor, as well as the patient’s knowledge, expectations and demand for care. If the patient has insufficient insight into the relation between his/her complaint and his work and/or psychosocial factors, and the demand for care is not work-related, GPTs find it difficult to address work in their practice. However, GPTs should not only follow the patient’s demand for care, but also investigate and provide information (about) the relation between the patient’s work and their complaints. GPTs can also inform patients about the role that GPTs can play in reducing absenteeism, presenteeism and return to work.

This study highlights that work is not always included in the scope of Dutch PTs; this was emphasized by the difficulty in recruiting participants for this study. Participants mentioned that work is a topic that can be difficult to address. An earlier qualitative study revealed that GPTs in Australia routinely initiated work discussions, but these therapists experienced difficulty in subsequently influencing changes at work [28]. In our study, some GPTs felt confident and able to address work-related factors, while others felt less competent. Although not many GPTs visited the patient’s workplace, participants recognized the importance of workplace visits. Others reported that a workplace visit had added value compared to the patient’s own description. One of the reasons GPTs did not perform a workplace visit was that they did not feel confident in this task [29], as also reported in our study. It seems that the use of photographs and (video) movies, as mentioned in our study, can provide added insight into the patient’s workplace.

Our participants experienced several barriers and areas that need facilitation with regard to the integration of work within GPTP. Similar barriers were found by Gosling et al. who investigated barriers/facilitators for return to work following a compensable injury [17]; they identified the following factors as barriers/facilitators impacting on GPTs ability to facilitate timely return to work: injured worker attitudes; the workplace; unified targets and positive approaches to care by all stakeholders; system delays; inappropriate certification of capacity; communication skills; and knowledge of the local compensation system [17, 30].

The lack of knowledge of GPTs was also seen as a barrier for addressing work within GPTP. Participants felt particularly uncertain about what kind of advice they are allowed to give patients, and about their knowledge related to workplace laws and regulations. Other topics requiring attention were: knowledge about work-related factors, how to address work-related factors, the workplace, workplace investigations, and knowledge about occupational healthcare providers.

### Strengths and limitations

Although a few studies have evaluated the experiences/perspectives of GPTs with regard to return to work [17, 29] or managing a condition in the context of work [28], to our knowledge ours is the first study to investigate how GPTs currently integrate work participation within their practice and how this could be facilitated. Moreover, this is the first study in the Netherlands to address this important topic. This study provides insight into the experiences, thoughts, beliefs and barriers of GPTs with regard to addressing work participation within their practice. A strength of this study is that we were able to include 30 participants of varying age (GPTs and OPTs), with different experiences and specializations, thereby ensuring a good reflection of the variety of GPTs in the Netherlands. Including GPTs and OPTs ensured interaction between both groups and provided more insight into their experiences, e.g. with regard to cooperation with each other. On the other hand, including GPTs and OPTs might have affected the expression of their opinions/responses, due to the presence of the category of GPT/OPT, and vice versa.

This study also has some limitations. The original aim was to include 5–10 participants (4 groups) in each focus group. However, due to difficulties with recruitment and last-minute cancellations, the number of participants per group ranged from 2 to 7. This implies that, in the smaller groups, less interaction was possible between participants. Due to this small group size and the fact that we did not purposively compose the focus groups, in three of the groups only GPTs or only OPTs participated. This could have influenced the outcomes of that focus group, i.e. that the opinion of GPTs or OPTs was not taken into account in these groups. However, in four groups both GPTs and OPTs participated and four groups fulfilled the criteria for mini focus groups [19]. A positive aspect of smaller groups is that this provided the opportunity to address some points more extensively.

This study may have suffered from selection bias. Since potential participants were recruited by news-mails and announcements. This implies that mainly GPTs already interested in work/occupation were included in this study, which could have influenced our results.

Participants were invited to talk about their opinions and experiences; however, they also gave their opinions about their GPT and OPT colleagues. For example, many participants felt that work participation could be better integrated, but that especially their colleagues could pay more attention to the patient’s work. Although most data are based on the participant’s own experiences, we should take into account that some data represent the participant’s opinions about others, which could be less reliable than data based on their own experiences. The results of the present study represent the experiences/opinions of the participants. Due to the varying background of the participants, we think that these results provide a reliable insight into the experiences of GPTs and OPTs in the Netherlands. However, because the healthcare system, more specifically the occupational healthcare system, may vary between countries not all results are generalizable to other countries.

### Future directions

The results of this study will be used to investigate (on a broader scale) how GPTs in the Netherlands currently integrate their patients’ work within GPTP and what their needs are. Moreover, the results of this study can be used to make adaptations in generalist physcial therapy educational programs and in EPFs, and to develop specific (online) courses. Moreover, it is recommended to develop an overview of work-related factors and tools which can be used by GPTs. Such an overview can be provided by an online toolbox. Also, facilitating a systematic approach for addressing work participation within GPTP seems valuable. In addition, the cooperation/networking between GPTs and OPTs, and knowledge about the OPT, should be enhanced. For this, a role might be played by the professional associations as well the individual therapists.

As a result of their study, Gosling et al. [17] made recommendations (including stakeholder education in compensation system processes, development of effective communication skills/strategies, and the use of online tools) to enable education and reduce the influence of factors that delay return to work. Based on these findings an online education program was developed [17, 30]. The users of this program responded positively and reported an improvement of their understanding of policy/procedures; moreover, early analysis of claims data suggests that this program also delivers positive results in terms of return to work rates [30]. Another study revealed that the addition of a brief early access vocational advice intervention [13], provided by a trained PT, for adults with musculoskeletal pain consulting a general practitioner was likely to lead to fewer days absent over the first 4 months and might have improved return to work self-efficacy in patients with MSDs who had work difficulties [31]. Therefore, it seems that training GPTs with regard to work-related factors (which can vary between countries and local situations) can result in better outcomes.

## Conclusions

This study provides insight into how GPTs integrate their patient’s work within GPTP and how integration of work within GPTP might be (better) facilitated. GPTs found it important to address the patient’s work within GPTP. However, most participants said that work is not addressed sufficiently. It was considered important to investigate whether or not work is involved as a causal factor or a barrier for recovery. Participants emphasized that it is important to address work-related factors in the subsequent treatment. Providing advice to patients with regard to work-related factors was considered important. There was a perceived lack of knowledge of GPTs with regard to knowledge about workplace laws and regulations, work-related factors, addressing work-related factors, the workplace, workplace investigations, and occupational healthcare providers (including the OPT). Cooperation between GPTs, OPTs and other occupational healthcare providers was considered insufficient and should be improved.

## Additional files

Additional file 1: Interview guide. (DOCX 13 kb)

Additional file 2: Overview of the identified main categories and underlying themes. (DOCX 53 kb)

## Abbreviations

MSDs: Musculoskeletal disorders
OPT: occupational physical therapist
PT: Physical therapist
PTs: Physical therapists
GPTP: Generalist physical therapy practice
GPT: Generalist physical therapist
GPTs: Generalist physical therapists
