# Supplementary material for: Physical therapists and importance of work participation in patients with musculoskeletal disorders: a focus group study
Source: BMC Musculoskelet Disord. 2017 May 16;18:196. doi: 10.1186/s12891-017-1546-9 (PMC5434575; doi:10.1186/s12891-017-1546-9)
Supplement: Supplementary file 1 — Interview guide. (DOCX 13 kb) [file 12891_2017_1546_MOESM1_ESM.docx]

**Additional file 1: Interview guide**

**Introduction**

The moderator provides a short introduction on the project with some background information

The moderator provides a short introduction about participating in a focus group

**Introduction: questions**

- Introduction of the participants (name, specialization, experience)
- Why do(n’t) you think it is important to address work participation within PT practice?
- What are advantages or disadvantages of integrating work participation within PT practice?
- How do you think patients feel about integrating work participation within PT practice?

**Way of working and cooperation**

- How do you integrate work participation within your practice (including history taking, physical examination, treatment (goals) and evaluation?
- With which occupational healthcare professionals do you work together?
- In what ways do you cooperate with them?
- When and why do you decide to refer a patient to an occupational healthcare professional?
- How do you refer them and to whom?

***Break***

**Improving integration of work participation within PT practice**

- How can PTs be facilitated to (better) integrate work participation within their practice?
- Which knowledge and skills do PTs need to adequately address work participation within their practice?
- What kind of support do you/PTs need to support the integration of work participation within PT practice?

**Closure**

- What was for you the most important topic addressed in this session?
- Did this session influence your opinion with regard to the subject of this session?
- Did you miss any topics with regard to the subject of this session?
