# Supplementary material for: Physical therapists and importance of work participation in patients with musculoskeletal disorders: a focus group study
Source: BMC Musculoskelet Disord. 2017 May 16;18:196. doi: 10.1186/s12891-017-1546-9 (PMC5434575; doi:10.1186/s12891-017-1546-9)
Supplement: Supplementary file 2 — Overview of the identified main categories and underlying themes. (DOCX 53 kb) [file 12891_2017_1546_MOESM2_ESM.docx]

Additional file 2: Overview of the identified main categories and underlying themes.
